# Mucosal immunization with *Lactiplantibacillus plantarum*-displaying recombinant SARS-CoV-2 epitopes on the surface induces humoral and mucosal immune responses in mice

**DOI:** 10.1186/s12934-023-02100-7

**Published:** 2023-05-09

**Authors:** In-Chan Hwang, Valerie Diane Valeriano, Ji Hoon Song, Marcela Pereira, Ju Kyoung Oh, Kyudong Han, Lars Engstrand, Dae-Kyung Kang

**Affiliations:** 1 Department of Animal Resources Science, Dankook University, Cheonan, 31116 Republic of Korea; 2 Department of Microbiology, Tumor and Cell Biology, Karolinska Institutet, Stockholm, 17165 Sweden; 3 Department of Microbiology, College of Science and Technology, Dankook University, Cheonan, 31116 Republic of Korea

**Keywords:** *Lactiplantibacillus plantarum*, SARS-CoV-2, Mucosal delivery vector, Oral vaccine

## Abstract

**Background:**

The use of probiotic lactic acid bacteria as a mucosal vaccine vector is considered a promising alternative compared to the use of other microorganisms because of its “Generally Regarded as Safe” status, its potential adjuvant properties, and its tolerogenicity to the host. Severe acute respiratory syndrome coronavirus 2 (SARS-CoV-2), which causes coronavirus disease (COVID-19), is highly transmissible and pathogenic. This study aimed to determine the potential of *Lactiplantibacillus plantarum* expressing SARS-CoV-2 epitopes as a mucosal vaccine against SARS-CoV-2.

**Results:**

In this study, the possible antigenic determinants of the spike (S1–1, S1–2, S1–3, and S1–4), membrane (ME1 and ME2), and envelope (E) proteins of SARS-CoV-2 were predicted, and recombinant *L. plantarum* strains surface-displaying these epitopes were constructed. Subsequently, the immune responses induced by these recombinant strains were compared in vitro and in vivo. Most surface-displayed epitopes induced pro-inflammatory cytokines [tumor necrosis factor alpha (TNF-α and interleukin (IL)-6] and anti-inflammatory cytokines (IL-10) in lipopolysaccharide-induced RAW 264.7, with the highest anti-inflammatory to pro-inflammatory cytokine ratio in the S1–1 and S1–2 groups, followed by that in the S1–3 group. When orally administered of recombinant *L. plantarum* expressing SARS-CoV-2 epitopes in mice, all epitopes most increased the expression of IL-4, along with induced levels of TNF-α, interferon-gamma, and IL-10, specifically in spike protein groups. Thus, the surface expression of epitopes from the spike S1 protein in *L. plantarum* showed potential immunoregulatory effects, suggesting its ability to potentially circumvent hyperinflammatory states relevant to monocyte/macrophage cell activation. At 35 days post immunization (dpi), serum IgG levels showed a marked increase in the S1–1, S1–2, and S1–3 groups. Fecal IgA levels increased significantly from 21 dpi in all the antigen groups, but the boosting effect after 35 dpi was explicitly observed in the S1–1, S1–2, and S1–3 groups. Thus, the oral administration of SARS-CoV-2 antigens into mice induced significant humoral and mucosal immune responses.

**Conclusion:**

This study suggests that *L. plantarum* is a potential vector that can effectively deliver SARS-CoV-2 epitopes to intestinal mucosal sites and could serve as a novel approach for SARS-CoV-2 mucosal vaccine development.

**Supplementary Information:**

The online version contains supplementary material available at 10.1186/s12934-023-02100-7.

## Background

Severe acute respiratory syndrome coronavirus 2 (SARS-CoV-2) is highly contagious, particularly in humans, and has resulted in a pandemic [1]. Many coronavirus disease (COVID-19) vaccines against SARS-CoV-2 have been developed and are widely used as preventive measures to contain its spread. However, many countries are overwhelmed by infection due to several factors such as low vaccine absorption, limited efficacy in some specific populations, and the continued emergence of novel mutations in SARS-CoV-2 [2]. In addition, genes involved in the origin and spread of novel mutations may further reduce the efficacy of current vaccines and therapies [2]. Epidemiological data on the survivors of SARS-CoV-2 infection have shown the lack of long-lasting protective antibodies against the virus; further research and alternative solutions are essential to diversify the potential prophylactic methods available [3–5].

The mucosal vaccine is a promising approach owing to its potential to induce both systemic and localized humoral and cell-mediated immunity [6]. Such long-term immunity would be achieved through highly regulated host-microbe interactions, cytokine signaling, and the immune system. Moreover, the mucosa and epithelial cell linings of the intestines, spleen, or lungs, which are key ports of SARS-CoV-2 entry, are important surfaces for preventing cellular infection by SARS-CoV-2 and other viral and bacterial pathogens with similar modes of entry [7]. This shows great potential in mass vaccination, such as the success of the oral polio vaccine and other licensed mucosal vaccines [8]. Compared to other host vectors, the use of lactic acid bacteria (LAB) as mucosal vaccine vectors is a promising alternative, owing to their “Generally Regarded As Safe” status, potential adjuvant properties, and tolerogenicity to the host. Furthermore, surface-exposed antigenic determinants of LAB have a high potential for recognition by the immune system [9].

As vaccine vectors, LAB mainly include *Lactococcus lactis* [10] and *Lactobacillus* species [11, 12]. The immunogenicity of *Lactiplantibacillus plantarum* (formerly *Lactobacillus plantarum*)-based vaccines is higher than that of *Lactococcus lactis* when orally administered to mouse models [9]. In addition, some *L. plantarum* strains are promising alternatives owing to their abilities to survive in gastrointestinal conditions and improving local and distal immune responses in vivo [13]. Recently, Wang et al. (2020) constructed a recombinant *L. plantarum* strain expressing the spike protein of SARS-CoV-2 and demonstrated its potential as a promising food-grade oral vaccine against SARS-CoV-2 [14]. Li et al. (2021) demonstrated that intranasal immunization with recombinant *L. plantarum* expressing the receptor-binding domain (RBD) of SARS-CoV-2 significantly increased mucosal immunoglobulin (Ig)A levels in mice [15]. Meanwhile, several studies [16, 17] also showed that industrially-amenable *Bifidobacterium* strains such as *Bifidobacterium infantis* can be considered as vaccine vectors also because reduced *Bifidobacterium* levels provide an important predictive factor of SARS-CoV-2 disease severity. However, further research is needed regarding mucosal vaccination against SARS-CoV-2 using probiotic bacteria. In addition, we determined other conserved epitope regions of the spike, membrane, and envelope proteins to explore potential alternatives for SARS-CoV-2 variants.

Our previous studies have demonstrated the potential of *L. plantarum* SK156 as an effective host for expressing bioactive substances in the intestine owing to its bile-responsive expression system [18, 19]. In this study, the possible antigenic determinants of the spike, membrane, and envelope proteins of SARS-CoV-2 were predicted and displayed on the surface of recombinant *L. plantarum* strains. Subsequently, the immune responses of the recombinant strains expressing different epitopes were compared in vitro and in a mouse model. This study aimed to determine the potential of *L. plantarum* expressing SARS-COV-2 epitopes as a mucosal vaccine against SARS-CoV-2.

## Materials and methods

### Bacterial strains and plasmids

The bacterial strains and plasmids used in the present study are listed in Table 1. *Escherichia coli* DH5α, grown in Luria-Bertani (LB) medium (Difco, USA) at 37 °C under shaking conditions (200 rpm), was used for plasmid DNA preparation and cloning. Ampicillin (100 µg/mL) was added if necessary. Lactobacilli were grown in de Mann, Rogosa and Sharpe (MRS) medium (Difco, USA) under stationary culture conditions at 37 °C. *L. plantarum* GK502 and SK156 were isolated from fermented Korean food. *L. plantarum* GK502 was used as a backbone plasmid to construct a surface display vector, and *L. plantarum* SK156 was used as a host for the surface display of the super folder green fluorescent protein (sfGFP) reporter or SARS-CoV-2 antigens. If necessary, 100 µg/mL ampicillin and 3 µg/mL erythromycin were used to treat *E. coli* and *Lactobacillus* spp., respectively.

**Table 1:** Bacterial strains and plasmids

Strains or plasmids	Characteristics	Source or reference
***Strains***		
*L. plantarum* GK502	Source of cryptic plasmid (NCBI accession number CP102362-CP102363)	This study
*L. plantarum* SK156	Recipient in transformation, erythromycin resistance negative	Chae et al. [16]
*L. johnsonii* PF01	Source of erythromycin resistance gene	Chae et al. [16]
*Escherichia coli* DH5α	Recipient in transformation for cloning	TaKaRa (Japan)
***Plasmids***		
pUC19	pBR322 replication origin, lacZ, Amp^r^	TaKaRa (Japan)
pGK02	Cryptic plasmid from *L. plantarum* GK502	This study
pUGK2	pUC19 with pGK02 fragment	This study
pUGK3	pUGK2 with Em^r^	This study
pUGK4	pUGK3 with surface display system	This study
pCB4270B-sfGFP	Source of reporter gene, super folder green fluorescent protein (sfGFP)	Jang et al. [20]
pUGK4-sfGFP	pULP4 with sfGFP gene from pCB4270B-sfGFP in surface display system	This study
pUGK4-Epitope	pULP4 with SARS-CoV-2 antigen in surface display system	This study

### Selection of SARS-CoV-2 virus antigen gene

**Fig. 1:**
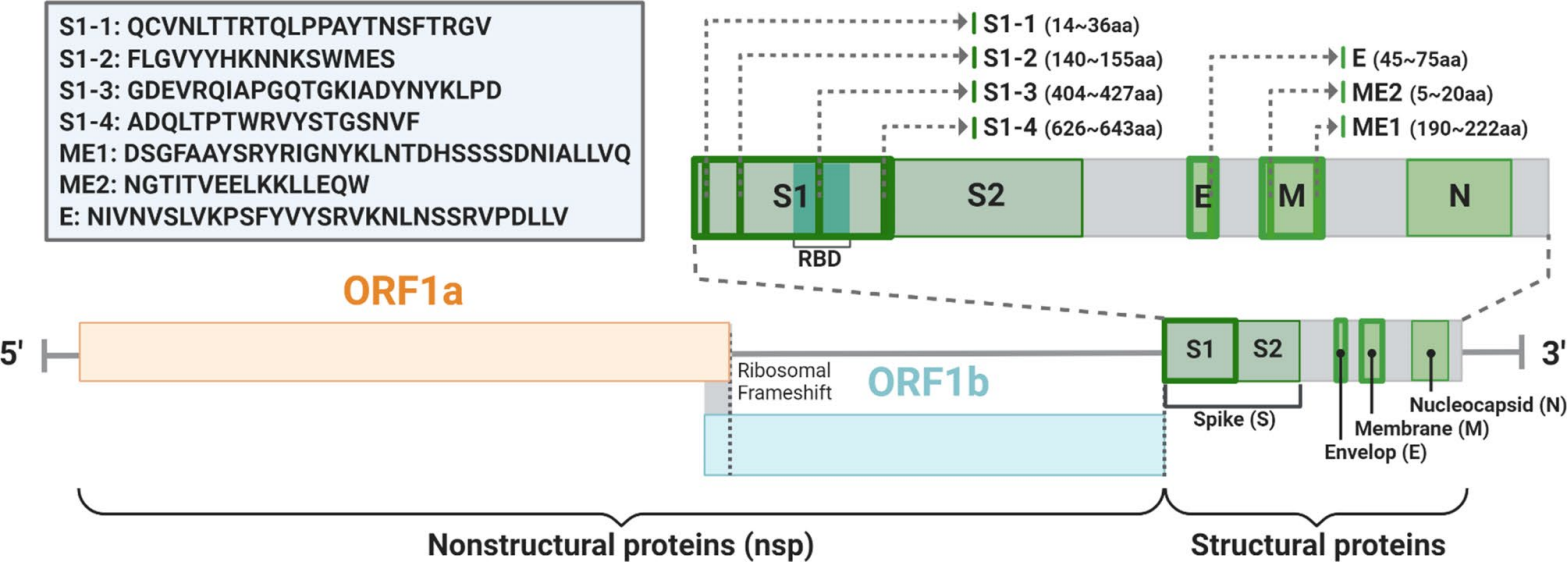
Severe acute respiratory syndrome coronavirus 2 (SARS-CoV-2) genome and conserved sequences of SARS-CoV-2 located in structural proteins (Additional Fig. 1)

Initially, SARS-CoV-2 sequences were acquired publicly using University of California, Santa Cruz (UCSC, https://genome.ucsc.edu/), and their genomes were compared to ensure the absence of mutations in the regions of interest. Nucleotide and protein sequences of these regions were obtained using UniProt (https://www.uniprot.org/) and the Expasy tools (https://www.expasy.org/). The Immune Epitope Database (IEDB, http://www.iedb.org/) was used to predict the B cell epitopes. Epitope selection was further optimized by assessing predicted protein folding of selected regions using IntFold (https://www.reading.ac.uk/bioinf/IntFOLD/) and assessing ligand-binding capacity and surface exposure, which were predicted together with the sequence of the anchoring protein of the vector system. Epitope selection was further honed by assessing predicted protein folding of selected regions using IntFold and assessing ligand binding capacity and surface exposure, which were predicted together with the sequence of the anchoring protein of the vector system (Fig. 1). The predicted epitopes were used for codon optimization based on *L. plantarum* and *E. coli* using Geneious Prime (2021.1.1 Java Version 11.0.9 + 11).

### Construction of surface display system

The primers used to construct the *E. coli-Lactiplantibacillus* shuttle vector and the surface display system are listed in Table 2. DNA fragments were amplified using polymerase chain reaction (PCR) using a PCR Thermal Cycler (TaKaRa Bio, Japan), and the reconstituted sequence was verified by DNA sequencing and restriction enzyme digestion. The PCR cycle involved pre-denaturation at 95 °C for 5 min, followed by 35 cycles of denaturation at 95 °C for 30 s, annealing at 55–65 °C for 30 s, and extension at 72 °C for 30 s. Subsequently, post-extension was performed for 10 min. DNA manipulation was performed according to the manufacturer’s instructions (TaKaRa Bio, Japan). *E. coli* plasmids were extracted using the Dyne Plasmid Miniprep Kit (DYNEBIO, Korea). The *Lactobacillus* plasmid was extracted after lysozyme (10 mg/mL) treatment with several modifications based on the experimental method of Sambrook et al. [21]. Both plasmids and DNA fragments were extracted on agarose gels using the NucleoSpin® Gel and PCR Clean-up Kit (MACHEREY-NAGEL, Germany).

**Table 2:** The primer sequences used for constructing the pUGK4 shuttle vector

Gene name	Primer sequence (5’ − 3’)	Restriction enzyme sites
pUC19	Forward: ATAGAGCTCTGGCGTAATCATG GTCATAGC Reverse: ATAGAGCTCAATTCACTGGCCGT CGTTTTAC	*Sac*I *Sac*I
pGK02	Forward: ATAGAGCTCGATCAACGGTAAA TCCGTTGGC Reverse: ATTGAGCTCGGTACCATATCCTG ACATTCTCTTTAC	*Sac*I *Sac*I, *Kpn*I
Erythromycin resistance gene (ErmR)	Forward: AATGGTACCGGATCCTTTCGCAG TAACTCTATTATCAAC Reverse: ATGGTACCGTCGACTGCAGGATC CTTATCTATTAAATAATTTATAG	*Kpn*I, *BamH*I *Kpn*I, *Sal*I, *Pst*I, *BamH*I
LDH1 promoter	Forward: ATAGTCGACCATTTGCACGAATT CTAATC Reverse: AATCTGCAGATACTTCCTTCCAT ATTAGTG	*Sal*I *Pst*I
Signal peptide (SP)	Forward: ATACTGCAGATGAAGAAAAATT TAAGAATCGTTAGCGCTG Reverse: ATAGCATGCGGGCCCTGATGAAC TTGCGTTAATA	*Pst*I *Sph*I, *Apa*I
Multi cloning site (MCS)	Forward: ATAGGGCCCGATATCGCCGGC Reverse: ATAACGCGTTCTAGACACGTGT CGCGA	*Apa*I *Mlu*I
Cell wall anchor(CWA)	Forward: AATGCATGCACGCGTAGGCCTA AGTCAGCTACTTTGC Reverse: ATAAAGCTTGGTACCAGGCCTTT AGTGGTGGTGGTGGT	*Sph*I, *Mlu*I, *Stu*I *Hind*III, *Kpn*I, *Stu*I
Super folder green fluorescent protein (sfGFP)	Forward: ATACTGCAGGGCCCATGTCAAA GGGTGAAG Reverse: AATCTGCAGCCGGCCTTGTATAA TTCATCCATACC	*Pst*I, *Apa*I *Pst*I, *Nae*I

The pGK02 backbone cryptic plasmid for the construction of a surface display vector was derived from the *L. plantarum* GK502 strain isolated from kimchi, a traditional fermented food. It was combined with pUC19 by cloning into the *Sac*I site; the erythromycin resistance gene (GCA_000219475.3) from *L. johnsonii* PF01, as a selection marker, was cloned into the *Kpn*I site. The bile inducible promoter of the lactate dehydrogenase 1 gene (GCA_000219475.3) [18] from *L. johnsonii* PF01 was PCR-amplified and *Sal*I and *Pst*I sites were cloned to express the target gene. The signal peptide and cell wall anchor of the surface layer protein A (SlpA) gene (Uniprot ID P35829) from *L. acidophilus* ATCC 4356 were amplified using PCR and located downstream of the target gene for cell wall anchoring of the target protein. sfGFP, a reporter gene confirming the expression of the surface display, and SARS-CoV-2 antigens were cloned into the restriction enzymes *Apa*I and *Nae*I at the multi-cloning site of the pUGK4 vector (Additional Table 1).

### Transformation

*E. coli* DH5ɑ transformation was performed using the heat shock method described by Sambrook et al. [21], and *Lactiplantibacillus* transformation was performed using the electroporation method modified from Kim et al. [22]. *Lactiplantibacillus* cells were grown in MRS broth containing 1% glycine at 37 °C until they reached early-log phase (OD_600nm_ = approximately 0.22). The cells were then washed twice in cold washing buffer (5 mM sodium phosphate, 1 mM MgCl_2_, pH 7.4) and resuspended in cold electroporation buffer (1 M sucrose, 3 mM MgCl_2_, pH 7.4). Further, approximately 2 µg of plasmid DNA was added to 50 µL of ice-cold cell suspension (approximately 10^9^ CFU/mL) in a disposable cuvette (Gene Pulser® Cuvete, 0.2 cm electrode gap; Bio-Rad, USA). The mixture was subjected to electroporation using GenePulser Xcell™ (BioRad, USA) under 2.0 kV, 200 Ω, and 25 µF capacitance conditions. After the pulse, the cell suspension was diluted up to 1 mL in MRS broth and incubated at 37 °C for 2–3 h. *E. coli* transformants were selected using LB agar supplemented with ampicillin (100 µg/mL), and *Lactobacillus* transformants were selectively grown on MRS agar supplemented with erythromycin (3 µg/mL).

### Western blotting and immunofluorescence assay

Target protein expression and cell surface display of *L. plantarum* SK156 transformants were confirmed using western blotting and immunofluorescence assays. The *L. plantarum* SK156 transformant was inoculated at 1% (v/v) in MRS medium containing erythromycin and incubated to the mid-log stage (OD_600nm_ = 0.4–0.5). Subsequently, 0.05% oxgall was added, and incubated at 37 °C for 6 h. The cells were harvested using centrifugation (15,000×g for 10 min at 4 °C) and washed twice with ice-cold 1× phosphate buffered saline (PBS, pH 7.4).

For western blotting, the washed cells were resuspended in ice-cold 1× PBS and disrupted using sonication (pulse 120 s, intervals: 10 s on, 15 s off; 38% amplitude). The cell lysate was mixed with 5× loading buffer and boiled in distilled water for 5 min for denaturation. The prepared protein sample was separated on a 12% glycine sodium dodecyl sulphate-polyacrylamide gel using an electrophoresis machine (100 V, Bio-Rad, USA) and transferred to a 0.45 μm polyvinylidene difluoride membrane (Bio-Rad, USA) through a transfer apparatus (400 mA, 90 min, Bio-Rad, USA) with 1× transfer buffer (25 mM tris, 200 mM glycine, pH 8.3). The membrane was rinsed thrice with 1× TBST (20 mM tris, 0.1% tween 20, pH 7.6) and blocked with 5% bovine serum albumin (BSA, R&D Systems, USA) in TBST at 25 ℃ for 1 h. Following this, the membrane was incubated with primary antibody (SARS-CoV-2 membrane, envelop, and spike protein antibodies, R&D Systems, USA) diluted (1:2000) in 2% BSA/TBST at 4 ℃, and gently shaken for 12 h. After washing thrice with 1× TBST, horseradish peroxidase (HRP)-conjugated secondary antibody (goat anti-mouse IgG, Invitrogen, USA) diluted (1:2000) in 2% BSA/TBST was added and shaken gently for 1 h. Subsequently, proteins were visualized using the ChemiDoc™ MP Imaging System (Bio-Rad) using Enhanced ChemiLuminescence reagent (Thermo Fisher Scientific, USA).

In addition, surface proteins of the transformants were identified using an immunofluorescence assay. A multi-well glass plate was prepared; 10 µL of 0.1% poly 7-L-lysine solution was added into each well and incubated for 1 h at 25 ℃. After washing the wells with distilled water and drying, 10 µL of transformant cells were added and incubated for 5 min. After fixing, the cells were washed with PBS containing 0.1% Tween 20 (PBST, pH 7.4) and blocked with 2% (w/v) BSA in PBST for 30 min at 25 ℃. After removing the blocking solution, 10 µL diluted (1:200) primary antibody (anti-GFP antibody, anti-His-tag antibody, or anti-SARS-CoV-2 antibody, R&D Systems, USA) dissolved in PBST containing 2% BSA was added and incubated in a humidified chamber overnight at 4 °C. The wells were washed with PBST buffer and subsequently incubated with 10ul of diluted (1:200) NL557-conjugated secondary antibody (NorthernLights™ Anti-mouse IgG-NL557, R&D Systems, USA) in PBST buffer containing 2% BSA for 1 h at 25 ℃ in the dark. The secondary antibody solution was then removed and washed with PBST in dark. For fluorescence microscopy, the bacterial cells were reconstituted using a 20% glycerol solution, mounted with a coverslip, and viewed using a microscope (ProgRes C10 plus with Intensilight C-HGFI, Nikon, Japan) with and without a filter at 570 nm.

### Macrophage cell

The RAW 264.7 murine macrophage cell line (ATCC, Manassas, VA) was grown at 37 °C in a humidified atmosphere of 5% CO_2_. The cell line medium was Dulbecco’s modified Eagle’s medium (DMEM): nutrient mixture F-12 (Ham, 1:1) with GlutaMAX™-I (DMEM:F12) (Invitrogen, USA), 10% fetal bovine serum (Atlanta Biologicals, USA), 5 ng/mL epidermal growth factor, 1% insulin-transferring selenium supplements, 1% penicillin-streptomycin (penicillin 10,000 U/mL, streptomycin 100 mg/mL; Invitrogen), and 15 mM HEPES. The cell culture media were replaced every 2 d, and the cells were passaged every 4–5 d by trypsinization with 0.25% trypsin-EDTA. To perform the in vitro experiments, RAW 264.7, cells were seeded at a suitable concentration as determined via 0.25% trypan blue viability staining. Immediately prior to use, confluent monolayers were washed 2–3 times with 1× PBS. The RAW 264.7 cells monolayer at 100% confluence (passages 21–45) were seeded onto 24-well plates and incubated until at least 90% confluence. Subsequently, *L. plantarum* SK156 transformants were adjusted to approximately 10^7^ CFU/mL, re-suspended in DMEM:F12 without antibiotics, and incubated with LPS-induced RAW 264.7 cells for 6 h. Following this, cell culture supernatants were collected and stored at -70 °C until further analysis.

### Animals, immunization, and Sample collection

Animal experiments were approved by the Dankook University Ethics Committee (DKU-20-053). Animals were handled and maintained under strict ethical conditions in accordance with international recommendations for animal welfare. For the immune response, 100 female, specific pathogen-free, 7-week old BALB/c mice (Raonbio, Korea) were purchased and acclimatized to laboratory conditions for 1 week. In the animal room, a 12-h light-dark period was maintained at 45–50% relative humidity and 22–25 °C, with *ad libitum* access to standard pellet diet containing 4 kcal/g of protein, 9 kcal/g of fat, and 4 kcal/g of available carbohydrate (2018 S Teklad Global 18% Protein Rodent Diet; Envigo, USA) and filtered tap water. Mice adapted to the laboratory conditions were randomly divided into 10 groups of 10 mice each and used for further experiments. Immunizations were performed with wild type *L. plantarum* SK156 or SK156 transformants, which were grown overnight in MRS medium with or without erythromycin (3 µg/mL) supplemented at 37 °C. Through oral feeding, 1.2 × 10^9^ CFU of wild or transformants in 100 µL PBS (pH 7.4) were fed on days 0–2, 14–16 (first booster), and 28–30 (second booster) for 3 consecutive days at 2-week intervals. To evaluate potential oral toxicity, body weights of the mice were measured and monitored weekly. All mice were euthanized using CO_2_ gas after day 35. For sampling, serum was collected from the cheeks of mice on days 0 (pre-immune), 21, and 35. Feces from each group were also collected, suspended in PBS containing 0.01 M EDTA-Na_2_, homogenized, and allowed to solubilize overnight at 4 °C. The suspension was then centrifuged, and the supernatant was collected. Clear extracts of all samples were collected by centrifugation and stored at -70 °C until further analysis.

### Cytokine detection

Sera from immunized mice and supernatants from RAW 264.7 cell lines were analyzed using 96-well maxi-binding immunoplates (R&D Systems, USA) to detect pro-inflammatory cytokines such as mouse tumor necrosis factor-alpha (TNF-α), interferon-gamma (IFN-γ), interleukin (IL)-6, and anti-inflammatory cytokines such as IL-10 and IL-4. Serum cytokine detection was performed according to the manufacturer’s protocol (R&D Systems, USA), and cytokine concentrations were determined using 2-fold dilutions of mouse recombinant TNF-α, IFN-γ, IL-4, IL-6, and IL-10. Standard curves and sample values generated for each cytokine were calculated spectrophotometrically at 450 nm. Thereafter, the data were analyzed using Graphpad Prism version 8.0 software (GraphPad Software, USA).

### Detection of antigen-specific IgG & IgA using enzyme-linked immunosorbent assay

Antibody immune responses to antigen-specific IgG or IgA from serum and fecal extracts were assessed using enzyme-linked immunosorbent assay (ELISA) for recombinant SARS-CoV-2 antigens. Recombinant SARS-CoV-2 antigens (100 µL of 10 µg/mL) expressed in *E. coli* were dispensed into each well of a 96-well plate (final concentration 1 µg/well) using carbonate-bicarbonate buffer (pH 9.6), and the plates were coated by incubation at 4°C overnight. The antigen-coated plate was washed with PBST and blocked by incubating for 1 h at 37°C in PBST containing 3% BSA. The plate was washed again with PBST, and 100 µL diluted immunized mouse serum (1:100) or fecal extract (1:10 dilution) was added to the wells and incubated at 37°C for 1 h. Subsequently, the plate was washed with PBST and 100 µL of IgG or IgA antibody (1:1000, Invitrogen, USA) was added to the wells. After incubation at 37°C for 1 h, the wells were washed with PBST. For antibody detection, 100 µL of 3, 3’, 5, 5’- tetramethylbenzidine was added to the well to induce a reaction in dark, and 50 µL of 0.5 N H_2_SO_4_ was added to arrest the reaction. The data were measured for absorbance, which was measured at OD 450 nm using an ELISA plate reader (SpectraMax M2e, Molecular Devices, USA).

### Statistical analysis

Statistical significance was determined using one-way analysis of variance (ANOVA) with Tukey’s tests using GraphPad Prism (GraphPad Software, USA). The results are presented as the mean ± standard error of mean. The statistical significance has been represented as follows: *p < 0.01, **p < 0.001, and ***p < 0.0001.

## Results

### Construction of surface display system

**Fig. 2:**
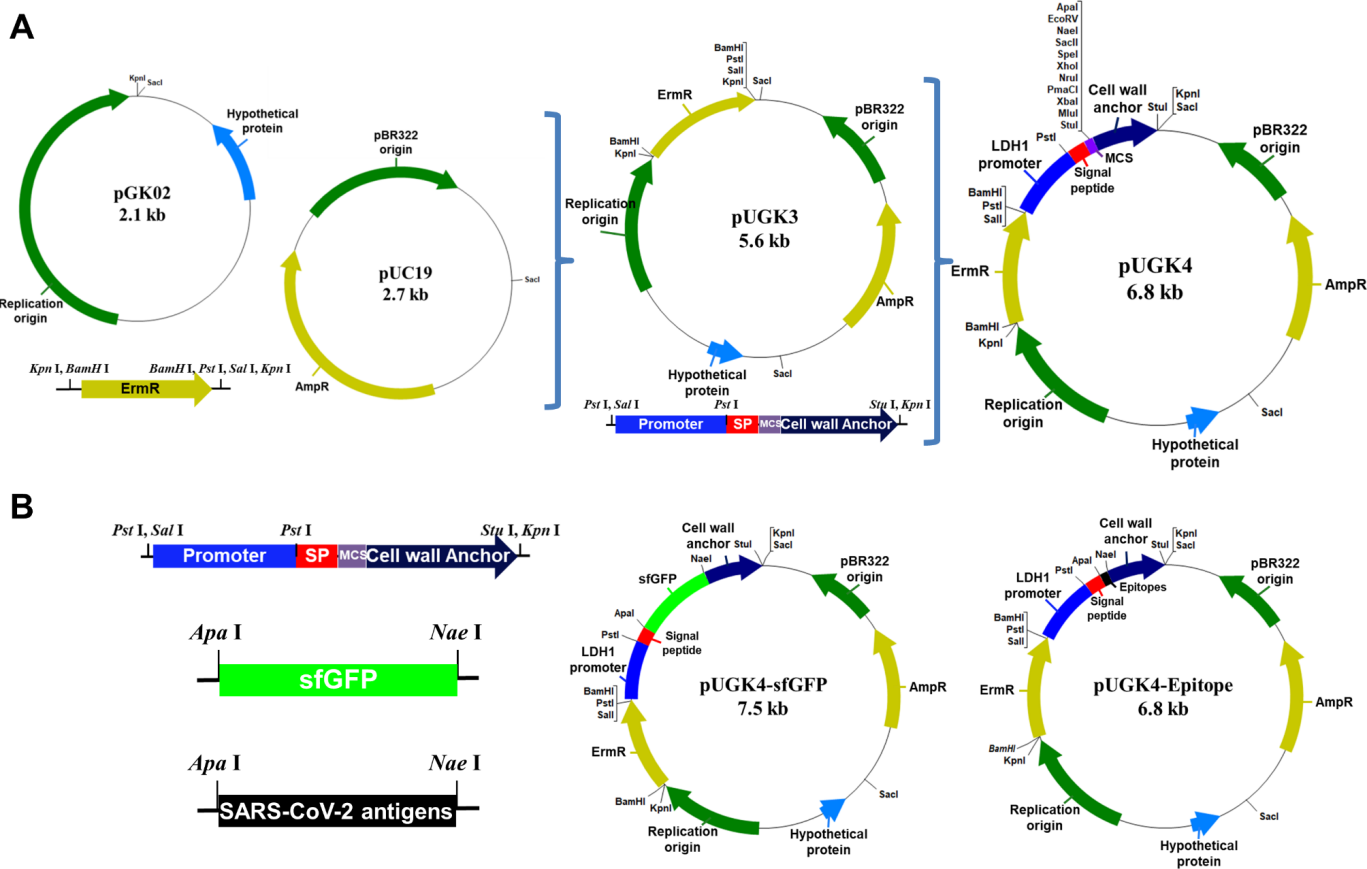
Schematic construct of *Escherichia coli-Lactobacillus* shuttle cloning vector pUGK4. (**A**) pUGK4 vector; (**B**) Surface display systems of super folder green fluorescent protein (sfGFP) and SARS-CoV-2 antigens

To produce a vector capable of expressing peptide epitopes on the surface of lactobacilli, the plasmid was constructed using pUC19 from *E. coli* cloning vector and pGK02 from *L. plantarum* GK502 (NCBI accession number CP102362-CP102363). After acquiring the publicly available SARS-CoV-2 sequences, seven putative B-cell linear epitopes (S1–1, S1–2, S1–3, and S1–4 from the spike S1 protein, ME1 and ME2 from the membrane protein, and E from the envelope protein) were chosen from the highly conserved regions of the SARS-CoV-2 genome. Codons of the predicted epitope sequences were optimized for expression in *L. plantarum.* Sequences of the seven epitopes are listed in Additional Table 2. The reporter gene sfGFP and seven epitopes of SARS-CoV-2 were cloned into the multiple cloning site of the pUGK4 surface display vector for cell surface expression on *L. plantarum* SK156 (Fig. 2, Additional Fig. 2).

### Confirming the surface display of SARS-CoV-2 antigens on ***L. plantarum***

**Fig. 3:**
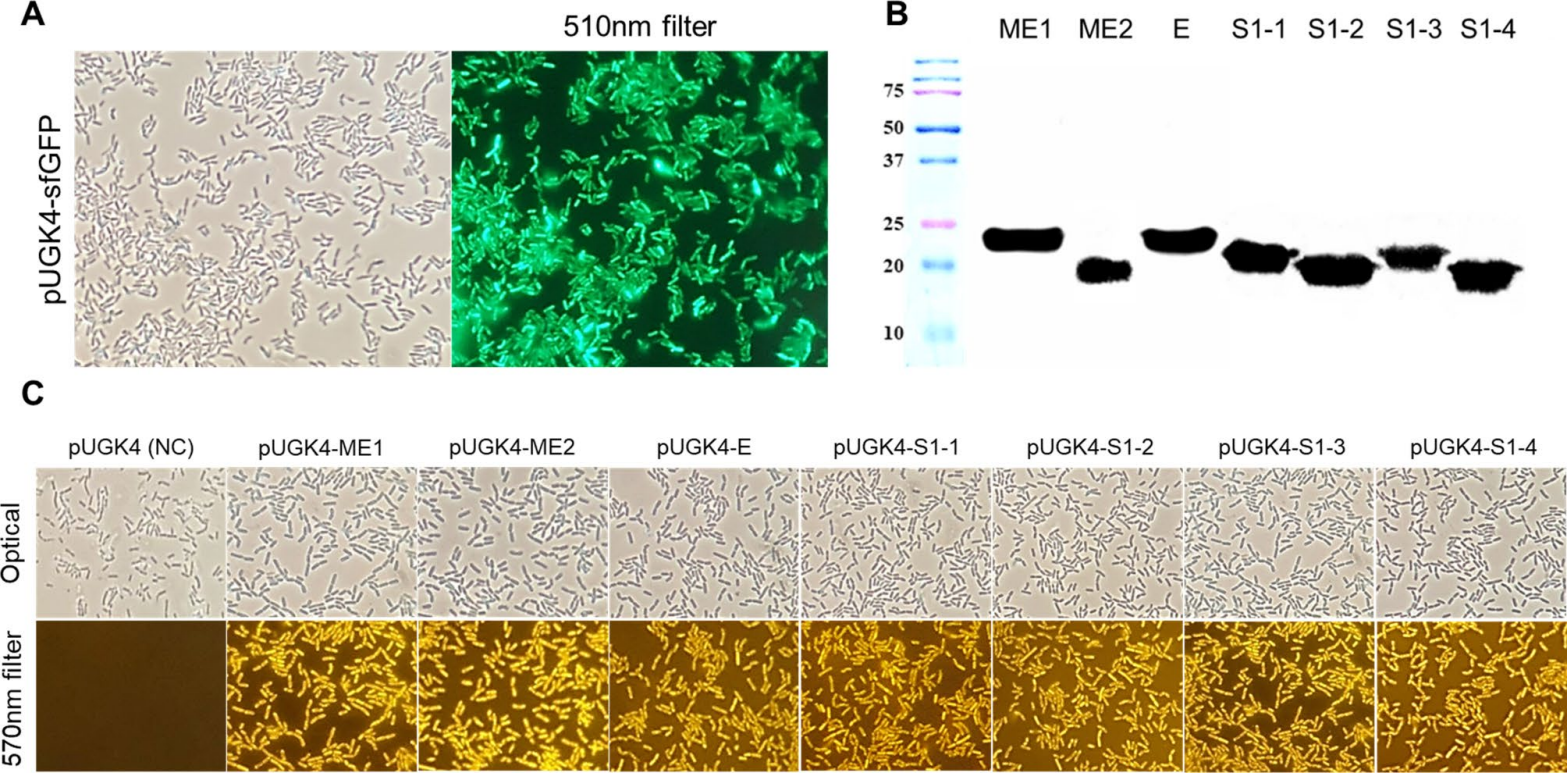
Confirmation for the expression of SARS-CoV-2 antigens and sfGFP on the surface of *Lactiplantibacillus plantarum*. (**A**) Surface display of sfGFP on *L. plantarum*. Cells were observed by using a fluorescence microscope to detect sfGFP fluorescence. (**B**) Expression of SARS-CoV-2 antigens. Western blot with SARS-CoV-2-specific polyclonal antibodies was used to detect the bands at 20–25 kDa. (**C**) Confirmation of surface display of SARS-CoV-2 antigens on *L. plantarum*, determined using immunofluorescence microscopy

To confirm the optimum functioning of the cell surface vector system, a sfGFP reporter gene was cloned into pUGK4 and transformed into *L. plantarum* SK156. Cell surface expression of the sfGFP reporter was observed at 510 nm using a fluorescence microscope. Green fluorescence was observed in the cells carrying sfGFP, in contrast to the control cells carrying only the vector, indicating optimum functioning of the pUGK4 vector system for cell surface display of the target protein in *L. plantarum* SK156 (Fig. 3A).

**Fig. 4:**
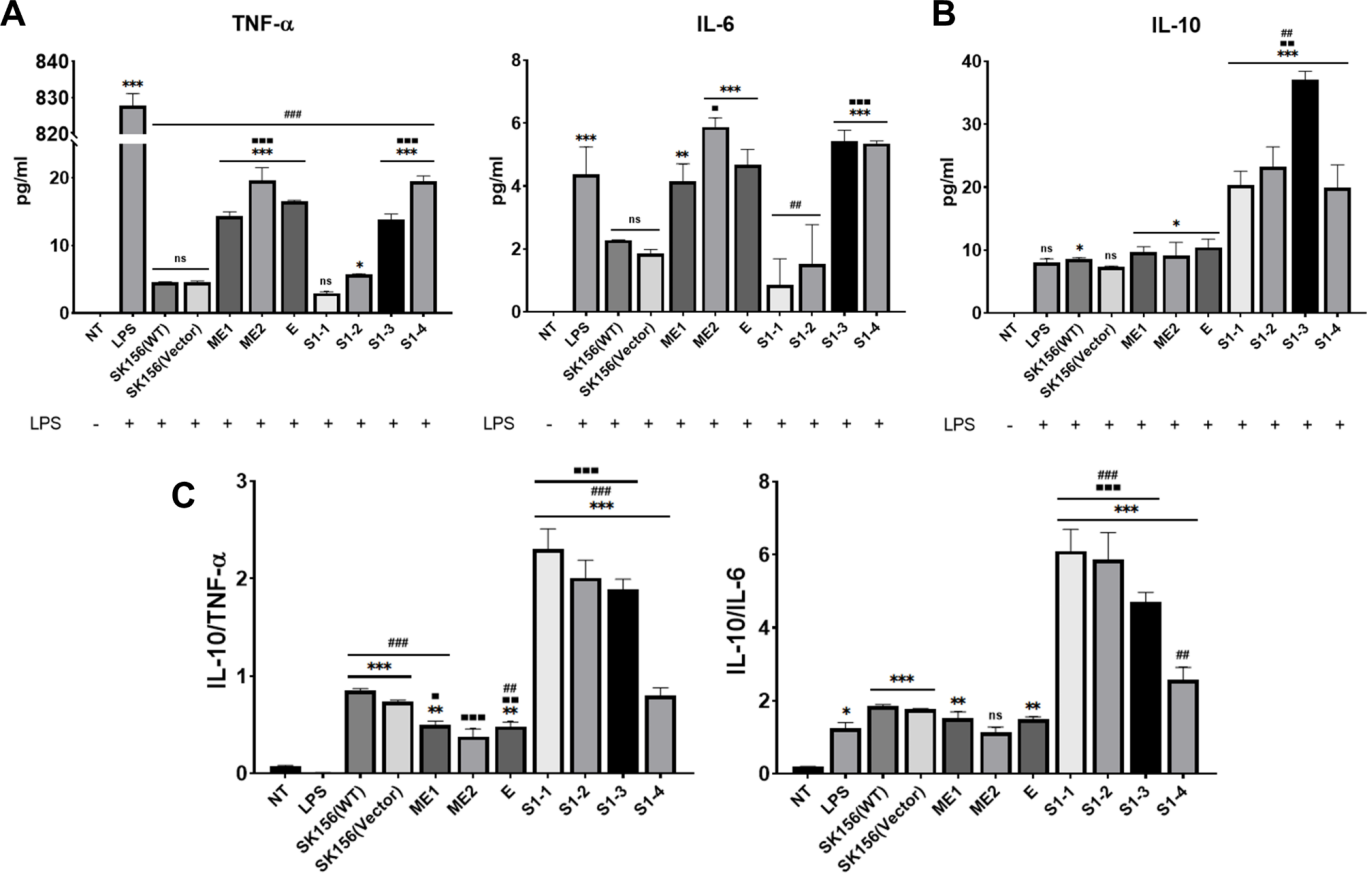
Tumor necrosis factor alpha (TNF-ɑ), interleukin (IL)-6, and IL-10 responses in RAW 264.7 cells stimulated with SARS-CoV-2 antigens. Concentrations of TNF-ɑ, IL-6, and IL-10 in the cell supernatants were detected using enzyme-linked immunosorbent assay. Error bars indicate the standard error of the mean. (**A**) Pro-inflammatory cytokines; (**B**) Anti-inflammatory cytokines; (**C**) Ratio of anti-inflammatory and pro-inflammatory. ‘*’ indicates the results of significance testing for the epitope groups versus the no treatment group (analysis of variance [ANOVA] test, *p < 0.01; **p < 0.001; ***p < 0.0001). ‘#’ indicates the results of significance testing for the epitope groups versus lipopolysaccharide (LPS) and ‘■’ indicates the results of significance testing for the epitope groups versus SK156 (wild type)

**Fig. 5:**
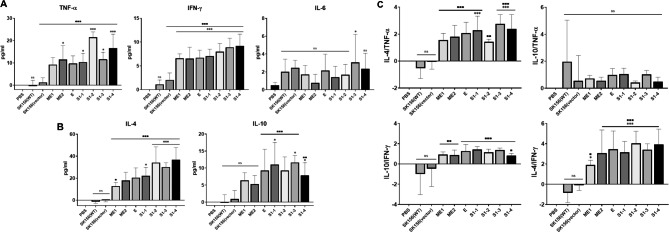
Cytokine responses of sera from mice immunized with surface displayed SARS-CoV-2 epitopes and wild type *L. plantarum* SK156. (**A**) Pro-inflammatory cytokines; (**B**) Anti-inflammatory cytokines; (**C**) Ratio of anti-inflammatory and pro-inflammatory. ‘*’ indicates the results of among the epitope groups, a group significantly increased compared to other groups (ANOVA test, *p < 0.01; **p < 0.001; ***p < 0.0001). ‘■’indicates the results of significance testing for the epitope groups versus the no treatment group

**Fig. 6:**
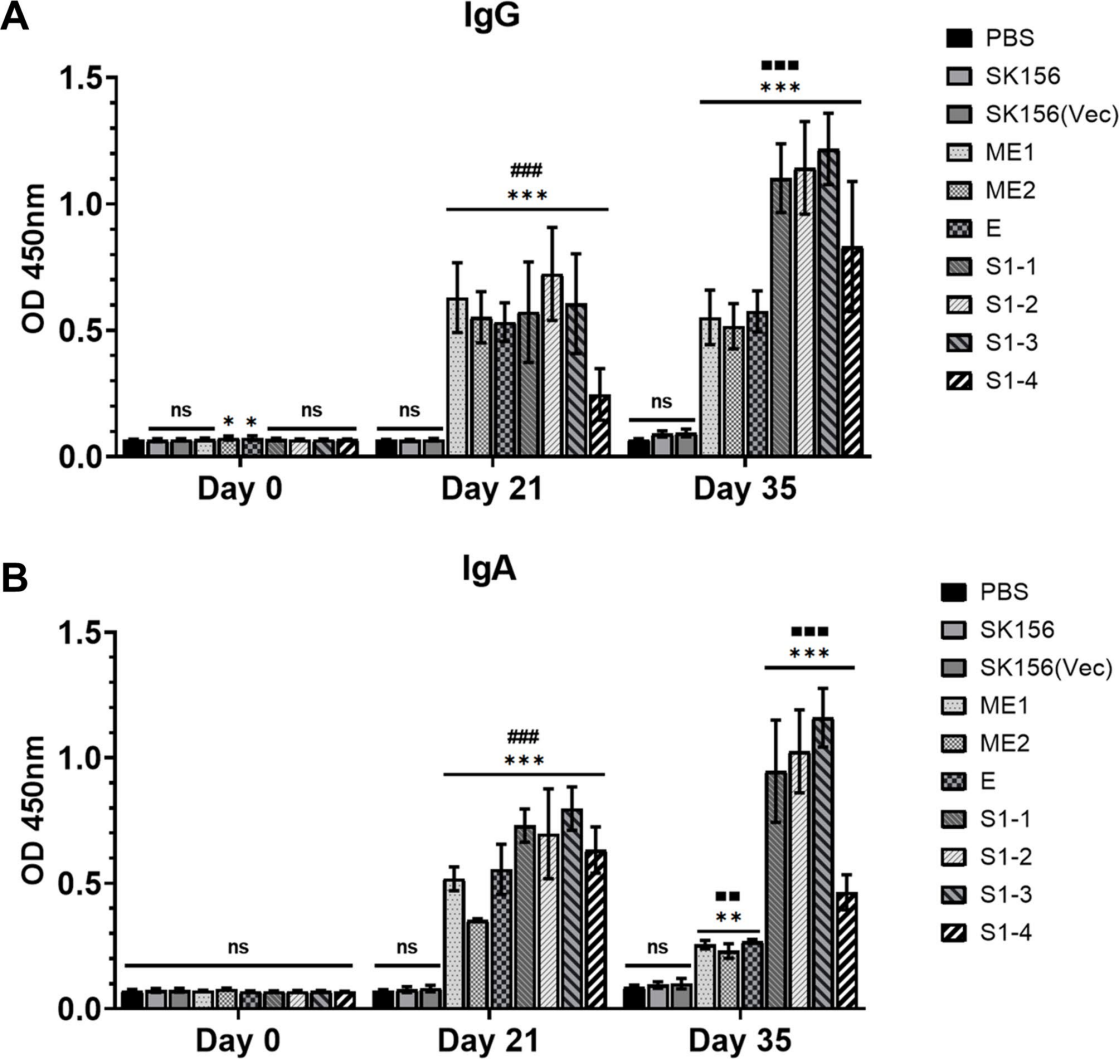
Antibody levels of the seven groups on days 0, 21, and 35 post first immunization. Immunoglobulin IgG in sera (A) and IgA in feces (B) prepared from mice orogastrically administered with surface displayed SARS-CoV-2 epitopes and wild type *L. plantarum* SK156, respectively. ‘*’ indicates the results of significance testing for the epitope groups versus no treatment group (ANOVA test, *p < 0.01, **p < 0.001; ***p < 0.0001). ‘#’ indicates the results of significance testing for the Day 0 groups versus Day 21 groups and ‘■’ indicates the results of significance testing for the Day 0 groups versus Day 35 groups

Further, surface expression of the SARS-CoV-2 epitopes was verified through western blotting using SARS-CoV-2-specific polyclonal antibodies. Bands corresponding to the SARS-CoV-2 epitopes fused with the cell wall anchor at 20–25 kDa were detected in cell lysates (Fig. 3B). Immunolabeling with specific antibodies is useful for detecting surface-exposed proteins [23]. Consequently, cells were probed with SARS-CoV-2 antiserum as a primary antibody and then visualized with HRP-conjugated goat anti-mouse IgG antibody. The presence of SARS-CoV-2 antigens on the surface of *L. plantarum* SK156 was confirmed using immunofluorescence microscopy at 570 nm, transformants carrying SARS-CoV-2 epitopes fluoresced brightly with the NL557-conjugated secondary antibody (Figs. 2 and 3C–8), whereas cells carrying the pUGK4 vector were not immunolabeled (Figs. 1 and 3C). Thus, SARS-CoV-2 antigens were successfully expressed on the surface of *L. plantarum* SK156.

### Cellular immune response of murine macrophage induced by recombinant ***L. plantarum***

To initially evaluated in vitro whether the SARS-CoV-2 antigens possess immunomodulatory activity. Murine macrophage RAW 264.7 cells were first stimulated with lipopolysaccharide (LPS) to induce the expression of pro-inflammatory cytokines and then treated with wild-type (WT) or recombinant *L. plantarum* expressing SARS-CoV-2 antigens. After treatment, the cytokines TNF-α, IFN-γ, IL-4, IL-6, and IL-10 were quantified using ELISA. The measured cytokines suggested the differentiation of macrophages into M1 or M2b type by LPS stimulation. IFN-γ and IL-4 were not detected in LPS-activated macrophages [24].

*L. plantarum* SK156 wild-type itself dramatically decreased pro-inflammatory cytokine (TNF-α and IL-6) levels in LPS-induced 264.7 RAW cells. These results indicated that *L. plantarum* SK156 has excellent anti-inflammatory activity. The levels of pro-inflammatory cytokines IFN-α, IFN-β, and IL-6 were significantly reduced in cells treated with the supernatant of *L. plantarum* Probio-88 strain [25]. In addition, the SlpA protein derived from *L. acidophilus* CICC 6074 [26], exopolysaccharide derived from *L. plantarum* L-14 strain [27], and *Aronia Melanocarpa* fruit fermented with *L. plantarum* EJ2014 [28] significantly reduced inflammation in an LPS-induced RAW 264.7 cell line model. Clinical data from infected patients show that SARS-CoV-2 is associated with a cytokine storm, which is one of the leading causes of death caused by the early elevation of systemic IL-10. Such stimulation is due to potential tissue damage related to the secretion of pro-inflammatory cytokines but results in immunosuppressive effects that prevent viral clearance [29, 30]. Notably, the same IL-10 induction by *L. plantarum* SK156 on activated macrophages may counteract potentially hijacked monocytes and macrophages which may be infected with SARS-CoV-2, thus promoting a more localized immunomodulation without dampening the potential for a systemic viral response [31]. Challenge models may be used to investigate this further.

Compared to the control groups, all recombinant strains, except for those expressing S1–1 and S1–2, significantly induced TNF-α and IL-6 expression (Fig. 4A). In contrast, IL-10 was greatly stimulated in S1–3, followed by that in the S1–1, S1–2, and S1-4 groups (Fig. 4B). The ratios of anti-inflammatory and pro-inflammatory cytokines, IL-10/TNF-α or IL-10/IL-6 were measured. The S1–1 and S1–2 groups showed the highest ratio, followed by that of the S3 group (Fig. 4C). In the context of an LPS-induced macrophage immune response, these results indicate that spike S1 protein epitopes have potential immunoregulatory effects, which may be because LPS-mediated inflammation is not aggravated to a level that may be detrimental to the host [32]. This is demonstrated by reducing TNF-α, but still maintaining continued IL-6 induction despite LPS-stimulated inflammation.

### Systemic cytokine changes induced by recombinant ***L. plantarum*** expressing SARS-CoV-2 epitopes in mice

Nine groups of mice were orally administered equal doses (approximately 10^9^ CFU/100 µL) of WT or recombinant *L. plantarum* expressing SARS-CoV-2 antigens. Three negative control groups, PBS, SK156 (WT), and SK156 with pUGK4 (vector only), were simultaneously maintained. After immunizing mice thrice over a 2-week period, serum was collected at 35 days post immunization (dpi) and analyzed for levels of pro-inflammatory (TNF-α, IFN-γ, and IL-6) and anti-inflammatory cytokines (IL-4 and IL-10) using ELISA (Fig. 5).

Regarding pro-inflammatory cytokines, all epitope groups showed a significant increase in TNF-α and the antiviral factor IFN-γ levels [14], but the IL-6 levels remained unchanged. The TNF-α level was highest in the S1–2 epitope, while there was no significant difference in IFN-γ levels among the antigen groups (Fig. 5A). Regarding anti-inflammatory cytokines, IL-4 levels were highest in spike proteins (S1–2, S1–3, and S1–4), but there was no significant difference among these groups. In contrast, compared to the control groups, the S1–1, S1–2, S1–3, and E groups showed high IL-10 levels, followed by those in the ME1 and ME2 groups. With increase in IL-4 levels compared to those of the pro-inflammatory cytokines TNF-α and IFN-γ, T helper (Th) 2 cell-biased immunomodulation may be achieved (Fig. 5B).

The ratios of the anti-inflammatory and pro-inflammatory cytokines are shown in Fig. 5C. There was no significant difference in the ratio of IL-10 to TNF-α among all the groups. However, IL-4/TNF-α, IL-10/IFN-γ, and IL-4/IFN-γ ratios were significantly increased in all the epitope groups compared to those in the control group, indicating immunoregulatory effects of all the epitope groups which potentially maintains IFN-γ signaling necessary for viral clearance.

### Humoral and mucosal immune responses induced by SARS-CoV-2 epitopes in mice

To assess antigen-specific humoral and mucosal immune responses induced by SARS-CoV-2 epitopes, sera and feces were collected from orally vaccinated mice on days 21 and 35 after immunization. Serum IgG levels significantly increased in all antigen groups at 21 dpi, a week after the first booster immunization (Fig. 6A). Among them, IgG levels in the S1–4 group were relatively lower than those of the other epitope groups. At 35 dpi, one week after the second booster immunization, IgG levels showed marked increase in the S1–1, S1–2, S1–3, and S1–4 groups. There was no substantial difference in the IgG levels between the S1–1, S1–2, and S1–3 groups. The ME1, ME2, and E groups showed relatively lower IgG levels than those of the spike protein groups.

To determine the local immune response, fecal IgA levels were measured (Fig. 6B). At 21 dpi, fecal IgA levels increased significantly in all the epitope groups. However, the boosting effect after 35 dpi was observed only in groups S1–1, S1–2, and S1–3, and there was no significant difference among these groups. These data suggest that the oral administration of SARS-CoV-2 epitopes into mice induced significant humoral and mucosal immune responses. Among them, S1–1, S1–2, and S1–3, from the spike protein, were more effective than the remaining epitopes, defining their potential antigenicity in a mucosal vector system.

These data suggest that the oral administration of SARS-CoV-2 epitopes can induce significant humoral and mucosal immune responses during immunization. Although the IgA levels at 35 dpi decreased compared to those at 21 dpi, the IgA level was significantly higher than that of the negative control, confirming the potential recognition of these epitopes (ME1, ME2, and E), but not to the same level as the S1 spike epitopes, except for S1–4, where the levels were lower than those in constructs S1–1, S1–2, and S1–3.

## Discussion

COVID-19 continues to spread globally. Current vaccination regimens have been effective in many countries in reducing disease severity and hospitalizations; however, they do not prevent transmission [33]. With many other countries still tackling these breakout infections, continuous improvement of available vaccines is still necessary, particularly to address the concerns of adverse events post-vaccination [34, 35] and steps closer towards neutralizing immunity.

A recent study comparing mucosal IgA and IgG responses in vaccinated participants clearly highlighted the importance of WT spike mucosal IgA production in lowering the risk of omicron breakthrough infection [36]. Similarly, it has been noted that current vaccination strategies induce only minimal mucosal sIgA responses in patients with no pre-exposure to COVID-19 compared to those in patients with a history of infection [37, 38]. Therefore, current research suggests that combining mucosal and systemic immune responses can achieve superior protection.

Moreover, vaccination is a pseudo-COVID-19 infection which trains the immune system [39]. Although vaccination has sometimes been associated cytokine release syndrome (CRS) in patients with cancer [40], correlations between mRNA vaccination and clinically relevant CRS diagnostic criteria remain unclear [41]. Benefit-risk ratios are still favorable for immunization. However, depending on the health status of the patients, serious risks such as a full-blown infection related to systemic inflammatory response syndrome and death due to an excessive cytokine response should still be considered [39, 42].

Thus, *L. plantarum* is a promising alternative vaccine carrier. Lactobacilli, including *Lacticaseibacillus rhamnosus, Lacticaseibacillus casei, Latilactobacillus sakei*, and *L. plantarum*, are relatively enriched in the upper respiratory tract, specifically in the anterior nares and nasopharynx of healthy individuals [42]. The oral consumption of commercial probiotics can contribute to niche colonization in the upper respiratory tract because of their anatomical connection via the nasopharynx, making them well adapted for possible development as a commensal mucosal prophylactic vector [43, 44]. In addition, *L. plantarum* strains are robust vaccine delivery systems and are well-characterized species, specifically in adhesion to mucosal surfaces and close interaction with host epithelial cells. This allows potential immunoregulatory activity [45–48] and antiviral properties against beta coronaviruses [49, 50], thus blocking SARS-CoV-2 infection at the point of entry [51, 52]. Furthermore, LAB vaccines can rapidly cope with mutated viruses. Thus, as soon as genetic information on the mutant virus is revealed, a LAB vaccine can be constructed and applied for vaccination in short periods.

LPS induction of naïve RAW 264.7, is a widely used approach to induce M1 macrophage polarization via Toll-like receptor 4 (TLR4) triggering of NF-kB and MAPK signaling cascades [53], and activating proinflammatory pathways. Proinflammatory cytokines, such as TNF-α and IL-6, but not IL-10, are upregulated. Alternatively, M2 macrophage (specifically the M2b subset) induction is also possible with LPS or IL-1 and an immune complex, performing a contrasting anti-inflammatory or immunoregulatory response [24]. In addition, although not investigated here, LPS-induced activation of macrophages induces angiotensin converting enzyme-2 (ACE2) expression via antibody-dependent Fc ligation or TLR4 [54]. This results in an increase in SARS-CoV-2 infection rates in M1 and M2 type alveolar macrophages and recapitulates potential inflammatory host states which may increase the risk of COVID infection, depending on certain cytokine or chemokine signaling cascades. Macrophage phenotypes may be reversible between M1/M2; however, lactobacilli are known to influence these activation cues, where an M2b macrophage phenotype leads to increased production of IL-10 (anti-inflammatory), IL-1, IL-6, and TNF-α, along with a reduction in IL-12 [55].

In this study, high IL-10 induction was observed with recombinant S1–3, expressing the RBD of the spike protein, along with induced levels of IL-6 and TNF-α, suggesting a potential immunoregulatory effect. S1–4 was similar, but with a lower IL-10 induction than that of S1–3. In contrast, S1–1 and S1–2 showed higher IL-10 levels but very low proinflammatory responses (IL-6 and TNF-α) after LPS induction, suggesting an M2-like macrophage phenotype. ME1, ME2, and E sustained the LPS-induced inflammatory response with low IL-10 induction.

The balance between Th1/Th2 responses is an important consideration in vaccine development [56]. COVID-19 severity is associated with CRS, with excessive elevation of IL-10 levels in critically ill patients [57, 58]. Although CRS is similar in SARS coronaviruses [59], the early response IL-10 elevation constitutes a potential mechanism which triggers a cytokine storm by a negative feedback loop response towards hyperinflammation and tissue damage [2]. Subsequently, this increases the endogenous IL-10 levels, allowing it to act as a pleiotropic immunostimulatory signaling molecule [60], such as that in autoimmune diseases and human cancers [57]. The possibility of its stimulating properties on immune cells such as CD4+, CD8 + T cells, and/or NK cells can increase the levels of inflammatory cytokines such as IFN-γ and IL-6 and markers such as C-reactive protein [57, 61, 62]. To balance this, a coordinated humoral and adaptive immune response may be achieved with mucosal immunization, where infection is controlled at the point of entry, particularly if these commensal bacteria or specific immune complexes can block access to ACE2 receptors by SARS-CoV-2 to prevent systemic infection at the mucosal lining of the lungs and intestines.

In our in vivo studies in BALB/c mice, both Th1 and Th2 responses were activated when only the recombinant lactobacilli strains were administered (no LPS). A significant increase in TNF-α and antiviral IFN-γ levels was observed without IL-6 overproduction. IL-6 levels are observed to be elevated in the cytokine storm caused by the available mRNA vaccine in humans, which may play a leading role in the adverse effects [63]. On the other hand, probiotic vaccination with the *L. plantarum* construct as shown in this study can provide protection without increasing IL-6. IL-4 levels were considerably high in the spike protein epitopes (S1–2, S1–3, and S1–4), providing a potential pathway towards B cell activation by Th2 CD4^+^ cell stimulation [64, 65]. Moreover, although the T cell subpopulation was not observed here, antigen-specific IgG and IgA showed a significant increase at 21 and 35 dpi, particularly for *L. plantarum* recombinant constructs expressing spike protein epitope fragments (S1–1, S1–2, S1–3, and S1–4). In contrast, the constructs expressing only epitope fragments from the membrane (ME1 and ME2) and envelope protein (E) were not sustained for IgG and IgA until 35 dpi. Nevertheless, induction of both humoral and cell-mediated immunity is promising.

Li and Colleagues [15] also produced an *L. plantarum*-based surface display construct expressing the RBD of the spike protein (LP18:RBD). Although specific IgG induction was not sustained due to varying factors such as formulation, route of administration, and immunization schedule, IgA induction was sustained in bronchoalveolar lavage fluid, nasal lavage fluid, and fecal samples [15]. Similar to most commensal bacteria, lactobacilli do not usually instigate inflammatory responses owing to their symbiotic relationship with the host, except with special species and strain-specific characteristics. Furthermore, an adaptive immune response is instigated in cross-talk hotspots, such as the M cells in the Peyers patches, where the specific pattern recognition receptors (PRRs) of macrophages and dendritic cells can recognize pathogen-associated molecular patterns to trigger the inflammatory response [66].

Similarly, SlpA in lactobacilli has been recognized to function in interacting with DC receptors via the Nod2 signaling pathway [55, 67] and is used as an efficient tool for surface display in *L. plantarum*. Similarly, a bile responsive system vector [18] can improve its role as a surface display vector when administered orally to its targeted area in the intestine. This background knowledge justifies PRR activation and allows the optimal utilization of such a *Lactobacillus* vaccine platform [55]. Nevertheless, other experimental data of *L. plantarum* in prospective trials for COVID-19 protection and potential boosting effects on vaccine-elicited immunity are promising [29, 68].

## Conclusions

In conclusion, we demonstrated that *L. plantarum* is a potential vector capable of effectively delivering SARS-CoV-2 epitope to mucosal sites. Especially, the expression of spike protein fragments in *L. plantarum* seems to be a promising immunoregulatory mucosal vaccine. This could be novel approach for the development of a mucosal vaccine against SARS-CoV-2. These oral and nasal vaccines are the next target for improved protection against SARS-CoV-2 owing to the lack of efficient protection against new mutant variants, including the omicron variants. Subsequently, we intend to further evaluate the construct in both in vitro and in vivo challenge model assays to confirm its potential in virus neutralization assays, mechanistic understanding in gut models, ability to influence T cell activation pathways, and possible correlation with the microbiome. Although LAB are safe microorganisms, further verification of the efficacy and safety of this delivery system for clinical application is also necessary.

## Electronic supplementary material

Below is the link to the electronic supplementary material.

**Additional file 1: Figure S1.** A 3D structure of the epitopes which corresponds to the SARS-CoV-2 protein using the I-TASSER & PyMOL programs. **Figure S2.** Construction of the E. coli-Lactobacilli shuttle vector. **Table S1.** Primer sequences used for the SARS-CoV-2 antigen candidates. **Table S2.** Proteins and optimized DNA sequences of SARS-CoV-2 antigen candidates.

## Data Availability

The data that support the findings of this study are in this published article and its supplementary information file and available from the corresponding author upon reasonable request.

## Code Availability

Not applicable for that section.
