# Supplementary material for: Mucosal immunization with Lactiplantibacillus plantarum-displaying recombinant SARS-CoV-2 epitopes on the surface induces humoral and mucosal immune responses in mice
Source: Microb Cell Fact. 2023 May 9;22:96. doi: 10.1186/s12934-023-02100-7 (PMC10169176; doi:10.1186/s12934-023-02100-7)
Supplement: Supplementary file 1 — Additional file 1: Figure S1. A 3D structure of the epitopes which corresponds to the SARS-CoV-2 protein using the I-TASSER & PyMOL programs. Figure S2. Construction of the E. coli-Lactobacilli shuttle vector. Table S1. Primer sequences used for the SARS-CoV-2 antigen candidates. Table S2. Proteins and optimized DNA sequences of SARS-CoV-2 antigen candidates. [file 12934_2023_2100_MOESM1_ESM.pdf]

<Supplementary data>

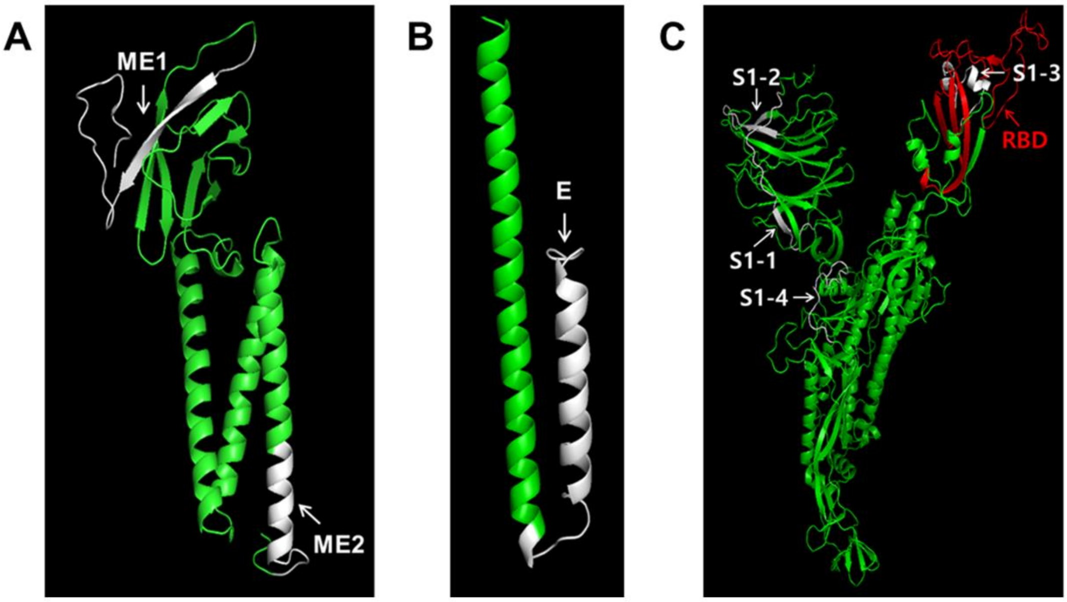

Supplementary figure 1. A 3D structure of the epitopes which corresponds to the SARS-CoV-2 protein using the I-TASSER & PyMOL programs. (A) membrane; (B) envelop; (C) spike.

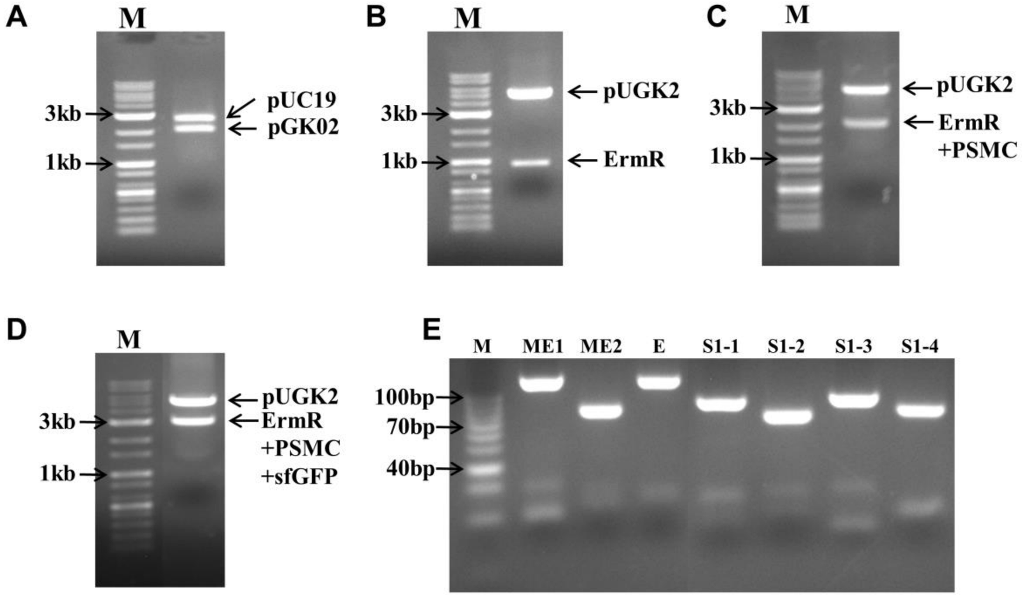

Supplementary figure 2. Construction of the *E.coli*-Lactobacilli shuttle vector. (A) pUGK2 (pUC19 with pGK02 fragment); (B) pUGK3 (pUGK2 with ErmR); (C) pUGK4 (pUGK3 with surface display system); (D) pUGK4-sfGFP (pUGK4 with sfGFP in surface display system); (E) polymerase chain reaction fragments of SARS-CoV-2 antigens for cloning into pUGK4.

Supplementary table 1. Primer sequences used for the SARS-CoV-2 antigen candidates

Gene name	Primer sequence (5’ - 3’)	Restriction enzyme sites
ME1	Forward: ATACTGCAGGGCCCATGGATAGTGGTTTTGCTG	*Pst*I, *Apa*I
	Reverse: AATGTCGACGCCGGCTTGAACTAACAATGCAATATTATC	*Sal*I, *Nae*I
ME2	Forward: ATACTGCAGGGCCCATGAATGGTACGATTACGG	*Pst*I, *Apa*I
	Reverse: ATACTGCAGGCCGGCCCATTGTTCTAATAATTTCT	*Pst*I, *Nae*I
E	Forward: ATACTGCAGGGGCCCATGAATATTGTTAATGTTAGT	*Pst*I, *Apa*I
	Reverse: ATACTGCAGCCGGCAACTAATAAATCTGGGAC	*Pst*I, *Nae*I
S1-1	Forward: ATAGGTACCGGGCCCATGCAATGTGTTAATTTAACGAC	*Kpn*I, *Apa*I
	Reverse: AATCTGCAGCCGGCAACACCCCGCGTAAAA	*Pst*I, *Nae*I
S1-2	Forward: ATTCTGCAGGGGCCCATGTTTTTAGGTGTTTACTATCA	*Pst*I, *Apa*I
	Reverse: ATACTGCAGCCGGCACTTTCCATCCAACTC	*Pst*I, *Nae*I
S1-3	Forward: ATACTGCAGGGCCCATGGGTGATGAAGTTCG	*Pst*I, *Apa*I
	Reverse: ATACTGCAGGCCGGCATCTGGTAACTTATAATTATAATCAG	*Pst*I, *Nae*I
S1-4	Forward: ATACTGCAGGGCCCATGGCCGATCAATTAAC	*Pst*I, *Apa*I
	Reverse: ATTCTGCAGGCCGGCAAAAACATTACTACCGG	*Pst*I, *Nae*I

Supplementary table 2. Proteins and optimized DNA sequences of SARS-CoV-2 antigen candidates

Name	Protein sequence	DNA sequence (5’-3’)	Function
ME1	DSGFAAYSRYRIGNYKLNTDHSSSSDNIALLVQ	GATAGTGGTTTTGCTGCCTATAGTCGGTATCGGATTGGTAATTATAAGTTAAATACGGATCATAGTAGTAGTAGTGATAATATTGCATTGTTAGTTCAA	Membrane protein
ME2	NGTITVEELKKLLEQW	AATGGTACGATTACGGTCGAAGAATTAAAGAAATTATTAGAACAATGG	
E	NIVNVSLVKPSFYVYSRVKNLNSSRVPDLLV	AATATTGTTAATGTTAGTTTAGTTAAACCAAGTTTTTATGTTTATAGTCGGGTTAAAAATTTAAATAGTAGTCGGGTCCCAGATTTATTAGTT	Envelop protein
S1-1	QCVNLTTRTQLPPAYTNSFTRGV	CAATGTGTTAATTTAACGACGCGGACCCAATTACCACCGGCTTATACGAATAGTTTTACGCGGGGTGTT	Spike protein
S1-2	FLGVYYHKNNKSWMES	TTTTTAGGTGTTTACTATCATAAGAATAACAAGAGTTGGATGGAAAGT	
S1-3	GDEVRQIAPGQTGKIADYNYKLP	GGTGATGAAGTTCGGCAAATTGCGCCAGGTCAAACGGGTAAGATTGCTGATTATAATTATAAGTTACCAGAT	
S1-4	ADQLTPTWRVYSTGSNVF	GCCGATCAATTAACGCCAACGTGGCGGGTTTATAGTACCGGTAGTAATGTTTTT	
